# The electronic structure and the formation of polarons in Mo-doped BiVO_4_ measured by angle-resolved photoemission spectroscopy†

**DOI:** 10.1039/c9ra01092k

**Published:** 2019-05-17

**Authors:** Mansour Mohamed, Matthias M. May, Michael Kanis, Mario Brützam, Reinhard Uecker, Roel van de Krol, Christoph Janowitz, Mattia Mulazzi

**Affiliations:** Institut für Physik, Humboldt-Universität zu Berlin D-12489 Berlin Germany mmulazzi@physik.hu-berlin.de; Departement of Physics, Faculty of Science, Assiut University 71515 Assiut Egypt; Institute for Solar Fuels, Helmholtz-Zentrum Berlin D-14109 Berlin Germany; OUT e.V. D-12555 Berlin Germany; Leibniz-Institut für Kristallzüchtung D-12489 Berlin Germany; Institute Functional Oxides for Energy-Efficient IT, Helmholtz-Zentrum Berlin D-14109 Berlin Germany

## Abstract

We experimentally investigated the electronic structure of Mo-doped BiVO_4_ high-quality single-crystals with synchrotron radiation-excited angle-resolved photoelectron spectroscopy (ARPES). By photon-energy dependent ARPES, we measured the bulk-derived valence band dispersion along the direction normal to the (010) cleavage plane, while the dispersion along the in-plane directions is obtained by angle-dependent measurements at fixed photon energy. Our data show that the valence band has a width of about 4.75 eV and is composed of many peaks, the two most intense have energies in good agreement with the theoretically calculated ones. A non-dispersive feature is observed in the fundamental gap, which we attribute to quasiparticle excitations coupling electrons and phonons, *i.e.* polarons. The determination of the polaron peak binding energy and bulk band gap allows to fix the value of the theoretical mixing parameter necessary in hybrid Hartree–Fock calculations to reproduce the experimental data. The attribution of the in-gap peak to polarons is strengthened by our discussion in the context of experimental transport data and *ab initio* theory.

## Abstract 2

Experimental investigation of the electronic structure of Mo-doped BiVO_4_ high-quality single-crystals with synchrotron radiation-excited angle-resolved photoelectron spectroscopy (ARPES).

## Graphical abstract

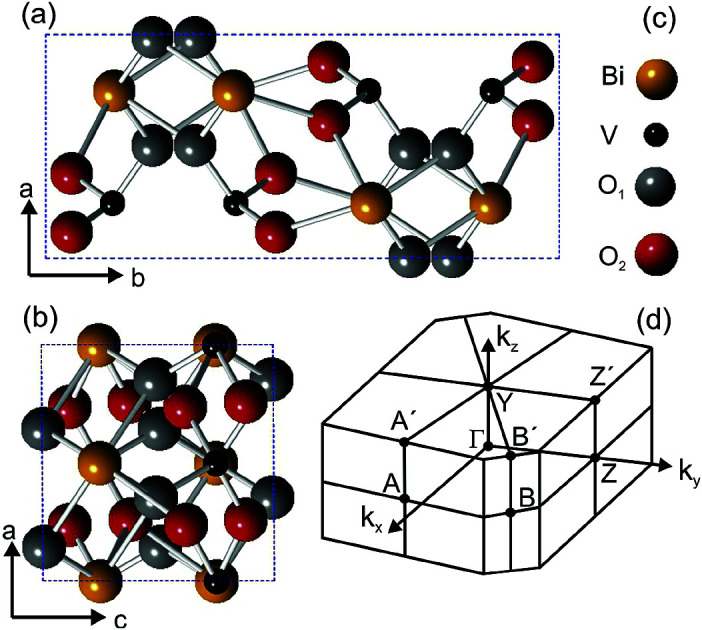

## 1 Introduction

Ternary metal-oxides are materials hosting a wide range of interesting properties, from either the fundamental or the applied point of view. For example, the family of ruthenium oxides shows metallic magnetism and spin-triplet superconductivity, observed in SrRuO_3_ and Sr_2_RuO_4_, respectively.^1,2^ Combining the Mott insulator LaAlO_3_ with the band insulator SrTiO_3_ in a heterostructure originates a two-dimensional electron gas^3^ at the interface, in which ferromagnetic^4^ and superconducting areas^5^ coexist. In applications, metal oxides are used as catalysts for solar water splitting applications^6^ and the material we are reporting on, BiVO_4_, is a very prominent representative of complex metal oxide semiconductors. BiVO_4_ is typically operated as a photoanode in combination with a second absorber, such as a silicon photovoltaic cell. While BiVO_4_ at present allows the highest metal-oxide-based conversion efficiency,^7^ it shows also efficiency-limiting factors such as sluggish electron transport,^8^ surface charge-carrier recombination^9^ and a band gap too large for high photocurrents under solar illumination. These limitations are directly related to the (surface) electronic structure of the material and investigating these issues may shed light on methods to increase the conversion efficiency. “Gradient doping” by tungsten significantly increases overall water splitting efficiencies,^10^ but the question remains to what extent relatively large amounts (*ca.* 1%) of dopants change bulk and surface electronic states, which could impact charge carrier recombination or electronic coupling to catalysts and the electrolyte. Consequently, a detailed knowledge of the surface electronic structure is crucial and while the literature on calculated electronic structures of BiVO_4_ is relatively extensive,^11–13^ there is, to the best of our knowledge, no experimental band structure available. Moreover, the coupling of electrons to the lattice degrees of freedom can originate unexpected many-particle effects, which can also hinder the charge transport. For instance, the formation of polarons can trap the electronic charge about a crystal site and therefore considerably reduce the mobility. The signature of polaron formation has been found in many oxide systems and recent theoretical calculations^14^ predicted their presence in BiVO_4_. Experimental transport^15,16^ and THz spectroscopy^17^ studies did indeed show evidence for them. However, none of these studies accessed directly the polaron energy, which is an important information: since the polaron energy lies in the band gap, it can contribute to photon absorption and, given the localization of the quasiparticle, it can fundamentally change the character of the band gap. Furthermore, polaron formation can limit the obtainable photovoltages of a photoelectrode material. Electron spectroscopies are particularly suited to measure the band structure, density of states (DOS) and quasiparticle excitations in solids, and the lack of experimental data can most probably be attributed to the poor conductivity of pure BiVO_4_. Introducing up to a few atomic percent of Mo or W, as also common in applied physics studies, enhances the electrical conductivity,^15,18^ which is also beneficial to solar water splitting applications^10^ and allows photoemission experiments. Though this is often referred to as “doping”, the term “dilute alloy” would be more appropriate, as also the optical properties of the single crystals are already significantly altered in this alloying region. We will, however, keep the term “doping” in the following due to common practice.

**Fig. 1:**
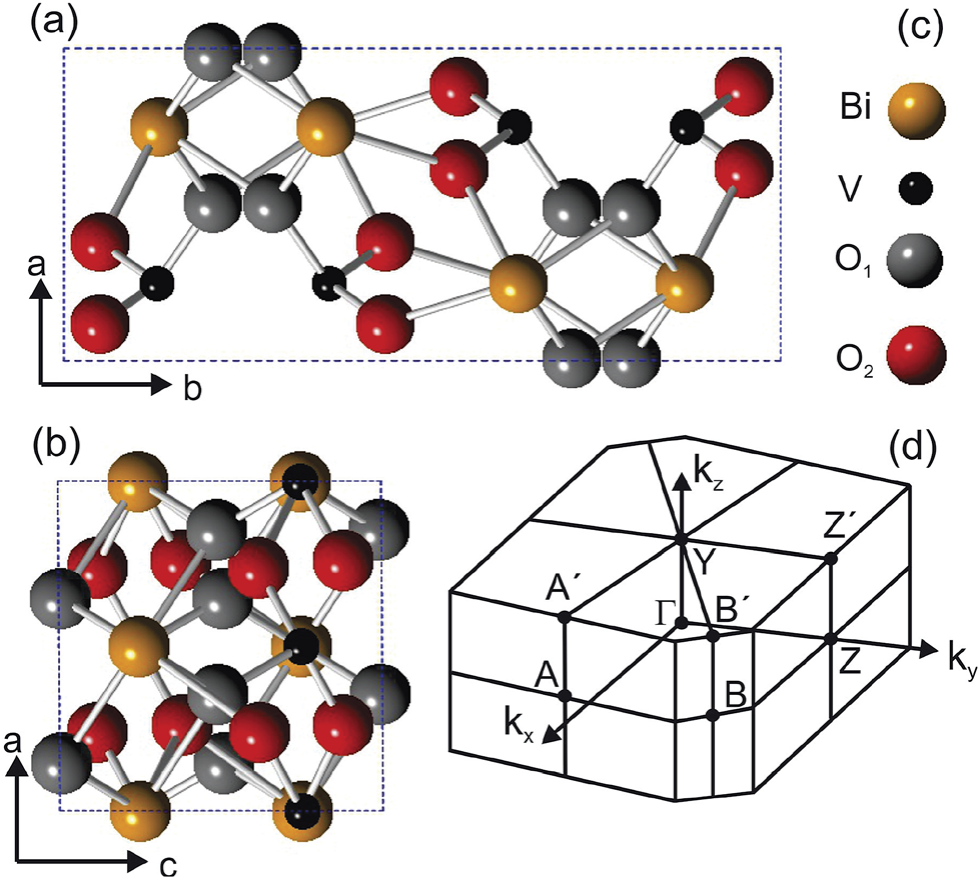
(a) and (b) Ball-and-stick model of the BiVO_4_ structure projected on the crystal planes containing the *a* and *b* lattice vectors (a) and the *a* and *c* lattice vectors (b). The color code of the atomic species is indicated in (c). (d) shows the shape of the Brillouin zone determined using the lattice parameter values given in the text.

BiVO_4_ exists in three different crystalline phases: monoclinic clinobisvanite,^19^ orthorhombic pucherite^20^ and tetragonal dreyerite.^19^ The monoclinic clinobisvanite is the most thermodynamically stable phase, has a monoclinic unit cell and X-ray diffraction data report^19^ lattice parameters lengths of *a* = 5.19 Å, *b* = 11.70 Å, and *c* = 5.09 Å. The angle between the *a* and *c* lattice vectors is *β* = 90.38°. The space and point groups are *I*2/*a* and 2/*m*, respectively. Fig. 1 shows the crystal structure of the BiVO_4_ unit cell projected along the *ab* and the *bc* planes.

The unit cell contains four Bi atoms, four V atoms and sixteen O atoms. The oxygen atoms are located at two different inequivalent sites, which are denoted O_1_ and O_2_. The unit cell vectors *b* and *c* are parallel to the *y*- and *z*-directions in the monoclinic structure, but since *β* is not exactly 90°, the *x*-direction lies at a δ*θ* = 0.38° from the *a* vector. The type of unit cell that we use is not the standard one, but it can be converted into the latter by a redefinition of the lattice axes, as indicated by Park *et al.*^21^ In this paper, we report on the experimental band structure of Mo-doped (1%) BiVO_4_ single-crystals measured by means of ARPES. The band structure has been measured along several directions of the surface Brillouin zone, shown in Fig. 1d, and for different photon energies. Energy dispersive bands have been observed along all high-symmetry direction and attributed to bulk states, while a non-dispersive state has been detected in the fundamental band gap. The latter is attributed to polaron formation at the molybdenum sites, which we understand by a photon-energy dependent analysis of photoemission cross sections. With this attribution, consistent with previous theoretical *ab initio* calculations and experiments, we have been able to determine the size of the parameter *α* used in hybrid calculations to mix in Hartree–Fock exchange.

## 2 Experimental

**Fig. 2:**
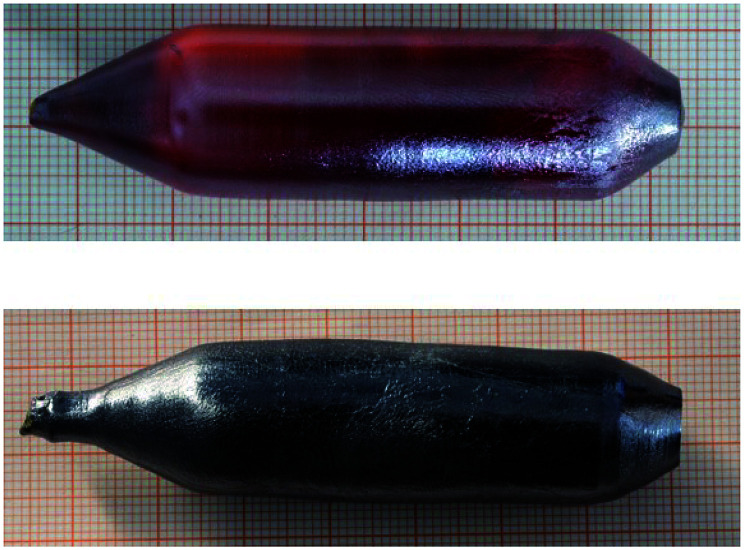
Two pictures of BiVO_4_ ingots obtained after the growth. The top panel shows an undoped crystal, which is semitransparent and of a reddish color. In the bottom part of the figure a crystal doped with 1% molybdenum is shown, which has a dark grey/black and a metallic luster, a clear sign of the modification of the bulk optical properties.

**Fig. 3:**
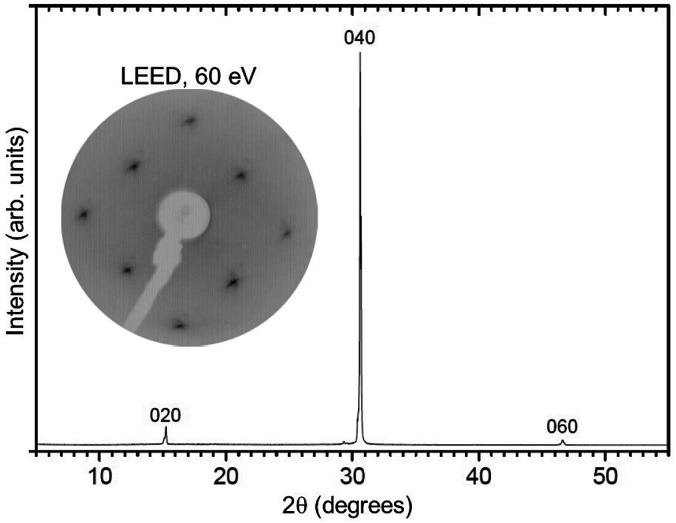
X-ray diffraction pattern for a BiVO_4_ single-crystal used for the ARPES investigations. The indices above the diffraction peaks indicate the diffraction planes. The inset at the top-left shows the LEED pattern of a UHV-cleaved BiVO_4_ crystal for which only the (1 × 1) spots are present, excluding surface reconstructions.

The investigated Mo-doped BiVO_4_ samples were cut from a bulk single-crystal grown by the Czochralski method. The starting oxides Bi_2_O_3_, V_2_O_5_ and MoO_3_ were of a minimum purity of 99.99%. To prepare the starting melt, the powders were dried, mixed in the stoichiometric ratio, sintered and finally isostatically pressed following standard procedures. The crystals were grown by the conventional Czochralski technique with RF-induction heating and automatic diameter control. Because of the low melting temperature of BiVO_4_ (1213 K), platinum crucibles were used as the melt container. This means that the crystals could be grown in air. To adjust the required temperature gradients, an active afterheater was installed above the crucible. The crucible dimensions were 40 mm in diameter and height; the afterheater was of the same diameter and 70 mm in height. An outer alumina ceramic tube filled with alumina granules provided thermal insulation. The seed crystal was won from spontaneously crystallized material. The pulling rate was 1.0 mm h^−1^ and the rotation rate was 10 rpm. After finalization of the growth, the crystal was cooled down to room temperature over 20 h. The resulting crystal (see Fig. 2) was of 77 mm in length and 18 mm in diameter, its high quality was confirmed by the rocking curve X-ray diffraction, see Fig. 3. Visually, the crystal appears metallic grey, as opposed to the pure BiVO_4_, which is orange, as shown in Fig. 2. The uniformity of the color on the ingot surface and inside the crystal, which was cut several times in air and cleaved in UHV, is a sign that the doping is of bulk origin and there is no segregation of the metallic Mo atoms to specific positions.

By X-ray photoelectron spectroscopy (not shown), we could determine that the amount of Mo is of the order of (0.5% ± 0.5%), indicating that the Mo concentration in the surface is quite close to (or slightly lower than) the nominal one expected form the ratio of the starting materials.

Prior to photoelectron spectroscopy, an electrical ohmic contact was fabricated on the back side of the samples by melting Sn to avoid sample charging. Once contacted, samples were glued onto a sample holder with silver epoxy. The clean (010) surfaces were obtained by cleaving under ultra-high vacuum (UHV) at a pressure of about 1 × 10^−10^ mbar and hence omitting any post-treatment such as annealing. The Fermi level position of gold film evaporated on the sample substrate was used as a reference for the binding energy of the ARPES spectra at the end of the respective experiment. The normalization of the spectral intensity was carried out using the mirror current at every photon energy applied. In addition, to check for possible sample charging under the photon illumination, experiments at different photon intensities were done with no observable shift in the photoemission spectra at room temperature, while evidence for charge-up was found when we decreased the sample temperature. At temperatures below 150 K, the spectra were shifted by several electronvolts and deemed unsuitable for analysis (shown in Fig. 8).

The *β* deviates only by δ*θ* = 0.43° from 90°, which would make the structure orthorhombic or quasi-tetragonal, given the small difference between the *a* and *c* lattice parameter. The small value of δ*θ* and the geometry used in the ARPES experiments justify the use of the non-standard unit cell. In fact, our experimental setup allows a rotation of the sample about two directions perpendicular to each other, namely parallel to the *a* axis and one perpendicular to it and consequently at an angle *β* from the *a* axis. This implies that the bands measured with ARPES by changing one angle lie exactly along the ΓA direction and along a direction at δ*θ* from the ΓZ direction. Therefore, our experimental bands could be directly compared to calculations carried out along the two above-mentioned direction, but unfortunately no comparison is possible to date. In fact on the one hand, it is impossible for us to measure along the second standard unit cell axis (lying at an angle of 44° from the rotation axis) and on the other hand because all theoretical calculations have hitherto been published in the standard unit cell.

The quality of the crystals as well as the orientation of samples for ARPES measurements were determined by low-energy electron diffraction (LEED) and X-ray diffraction (see Fig. 3). For the LEED measurements, the sample was cleaved in a preparation chamber at a base pressure of 10^−8^ mbar and transferred to the LEED chamber (10^−10^ mbar), equipped with a SPECS Er-LEED.

The high-resolution ARPES measurements were carried out at the synchrotron radiation source BESSY II of Helmholtz-Zentrum Berlin. All the photoemission experiments were performed at room temperature on the 5m-NIM beamline,^22^ delivering photons in the 8–50 eV energy range and equipped with an SES 2002 hemispherical electron analyzer. For the data presented here, the energy and wavevector resolutions were determined to be 20 meV and 0.1 Å^−1^, respectively.

## 3 Results

The powder color of the BiVO_4_–MoO_3_ studied here is of greenish yellow (yellow for the pure material). The experimental values of the lattice parameters of our crystals from X-ray diffraction (Fig. 3) are *a* = 5.18 Å, *b* = 11.70 Å, *c* = 5.10 Å, and the angle *β* = 90.43°, which are in good agreement with the data in ref. 19. The appropriate determination of the Brillouin zone's (BZ) shape is important for the electronic structure investigation, in particular for monoclinic structures, since the shape of the BZ depends on the actual length of the lattice parameters.^23^ With the values of the above-mentioned lattice parameters for a simple monoclinic system, we obtained a BZ with the shape shown in Fig. 1d.

The LEED pattern shows an unreconstructed surface with the *a* and *c* lattice vectors in the surface plane, confirming that the BiVO_4_ single-crystals are oriented with the (010) parallel to the surface and consequently the *b* vector perpendicular to it.

**Fig. 4:**
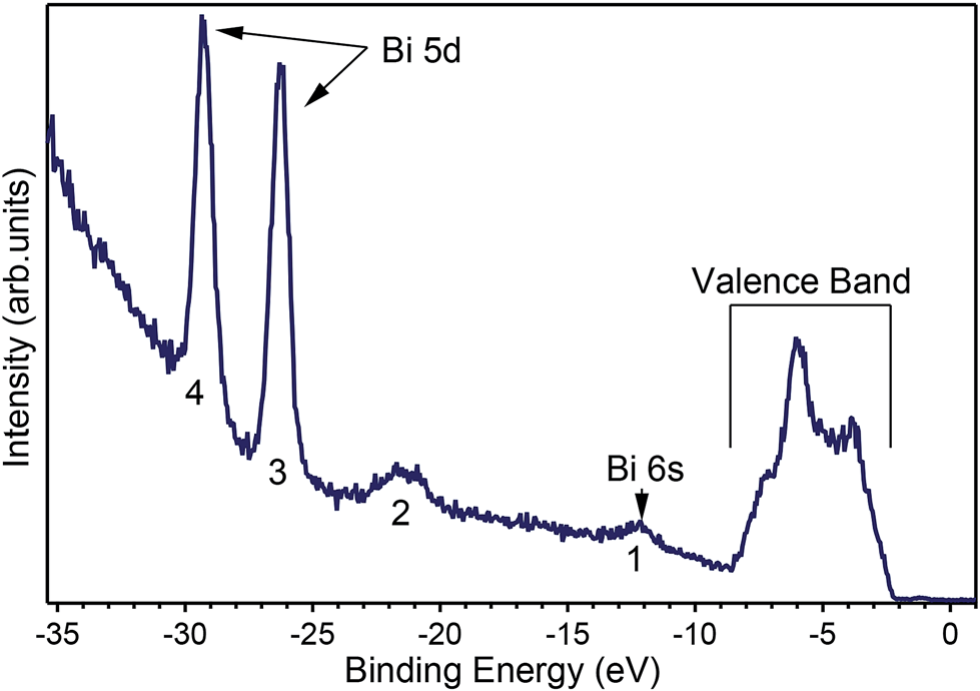
Angle-integrated spectrum measured at *hν* = 50 eV showing the peaks for (1) Bi 6s, (2) O 2s, (3) and (4) the Bi 5d states split by the spin–orbit coupling and the valence band occupying the energies from 2 eV to 10 eV below the Fermi level, which is the zero of the scale.

A number of recently published papers reported the calculations of the theoretical band structure of BiVO_4_ based on density functional theory and a base-centered monoclinic structure.^11–13^ To the best of our knowledge, however, no experimental band structure data of single-crystals is available to compare to, probably because of the strong insulating character of the pure material. Due to the scarcity of experimental data we start presenting in Fig. 4 an angle-integrated photoemission spectrum taken with *hν* = 50 eV, a photon energy high enough to allow for the measurement of the valence band as well as the shallow core levels of the material.

**Fig. 5:**
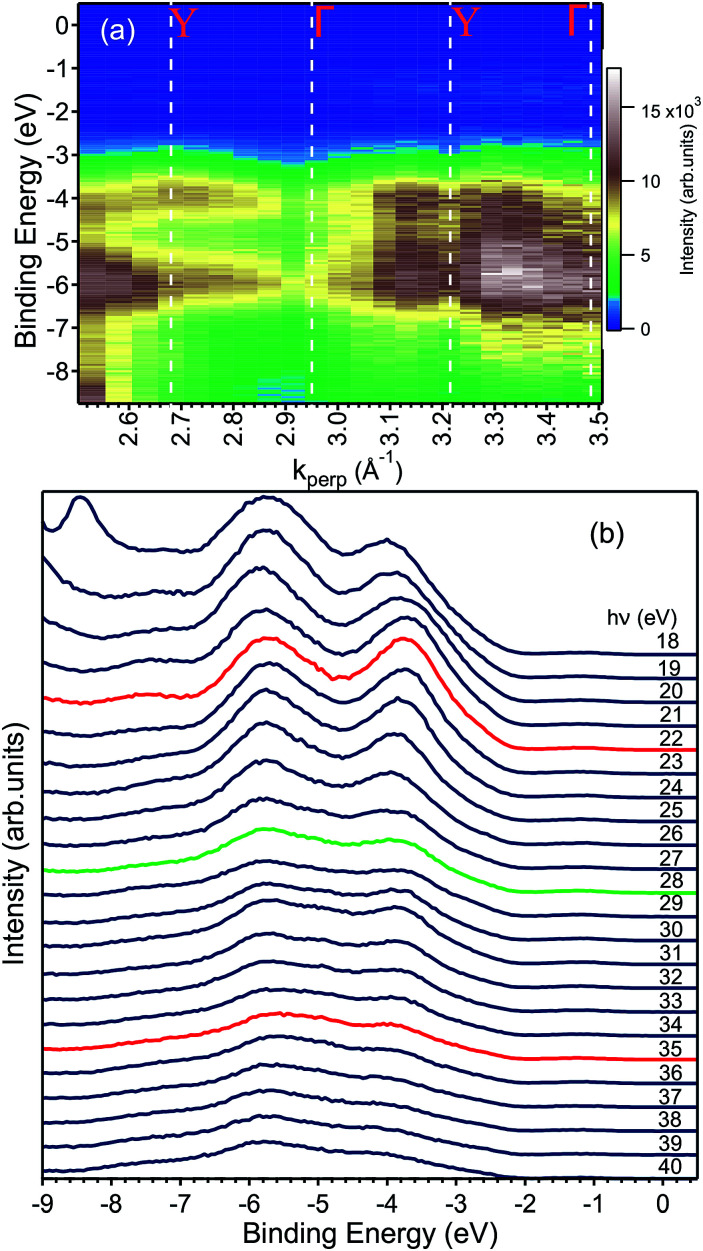
(a) ARPES map showing the valence band dispersion along the ΓY direction with the values of *k*_⊥_ corresponding to high symmetry points of the bulk BZ. (b) The same data shown in (a) are here represented as EDCs. The peak at −8.5 eV in the *hν* = 18 eV data is an artefact and no electronic state. Careful inspection −2 eV and the Fermi level reveals an in-gap peak, which is presented in Fig. 7 and discussed in the text. The EDCs marked in red indicate the spectra taken at the Y point of the Brillouin zone, while the green one to that taken at the Γ point.

Fig. 4 shows the valence band at lower binding energies below the Fermi level. Furthermore, four characteristic peaks labelled 1, 2, 3 and 4 are observed at binding energies of 12.2 eV, 21.5 eV, 26.2 eV and 29.2 eV, respectively. The least intense peak 1 corresponds to Bi 6s core levels, peak 2 to oxygen 2s core levels, while the most intense sharp features 3 and 4 have been assigned to the Bi 5d emission. The appearance of two peaks for the Bi 5d core levels is due to spin orbit coupling because of the final states effects in the photoemission process. The attribution of peak 1 in Fig. 4 to the Bi 6s states is in agreement with reference data for atomic binding energies as well as with theoretical calculations for the material. In fact, the difference between the energies of the experimental peak and valence band maximum is 9.8 eV, in good agreement with the theoretical calculations^11–13^ and with recently published experimental data.^24^ The ARPES spectra at normal emission reported in Fig. 5a indicate that the spectrum at 2.7 Å^−1^ (corresponding to a photon energy of 22 eV) is the one for which the valence band reaches its maximum. Thus, we extracted the band gap by fitting a line to the Energy Distribution Curve (EDC) in the energy range (−3.5, −2.5) eV (around the inflexion point of the EDC) and obtained a value of 2.55 eV, which is larger than the theoretical value of 2.4 eV of BiVO_4_.^25,26^ Since photoemission is not capable to measure the empty conduction band, strictly speaking, our experimental value of the gap must to be considered a lower bound for the “true” band gap. Assuming that the n doping provided by the 1% of molybdenum is high enough to place the Fermi level 100 meV below the conduction band, then the experimental value of the band gap lies between 2.55 and 2.65 eV.

We measured the dispersion of the valence states as a function of the momentum-direction perpendicular to the surface, *k*_⊥_ corresponding to the ΓY direction, by changing the photon energy and as a function of one in-plane direction (ΓZ) by varying the emission angle at fixed photon energy. The calculation of reciprocal space locations has been done using the standard formulae of the free-electron final state model.^27^

Fig. 5a shows the data along the ΓY high-symmetry line (see Fig. 1), which have been taken with photon energies from 18 to 40 eV. A number of dispersive bands can be observed, whose relative intensities vary as a function of the excitation energy due to matrix element effects.

The photon energy was converted to *k*_⊥_, following general practice,^27^ by using the value for the inner potential obtained in LEED experiments. Here, the intensity maxima of the main Bragg peak are determined as a function of energy (see ESI†) and the inner potential, *U*_0_, is treated as a fitting parameter,^28^ which results in *U*_0_ = (17.8 ± 3.5) eV. This value of *U*_0_ obtained from the LEED experiments has been used to convert the photon energy to *k*_perp_ in the ARPES dataset. The *k*_⊥_-dependent ARPES band dispersion is shown in Fig. 5 in the form of a colour map and in Fig. 5b as EDCs. Dispersion maxima and minima can be observed around 2.68 and 2.95 Å^−1^ corresponding to the high symmetry points Y and Γ, respectively. This assignment of the high-symmetry points is correct if either the LEED value of the inner potential or the symmetry in the ARPES dispersion is used. Since the distance between two Γ points is 0.537 Å^−1^, the ARPES spectra along ΓY show the *k*-space region across the fifth BZ whose edges are reached at 22 and 35 eV, respectively. Although not readily visible in Fig. 5a, the periodicity of the band energy is present in the Fig. 5b. The reason for the seeming lack of dispersion in the intensity maps lies in the dependence of the photoemission matrix elements on the photon energy, which enhances or suppresses some bands depending on the final state available.

To obtain the width of the valence band, the spectrum at the Γ point (22 eV) was fitted by a total of six Gaussian peaks located at −1.20, −2.70, −3.67, −5.19, −5.87 and −7.45 eV (not shown). The difference between the topmost and bottommost binding energies (−7.45 and −2.70 eV) provides a value of 4.75 eV, a reliable estimate of the valence bandwidth.

**Fig. 6:**
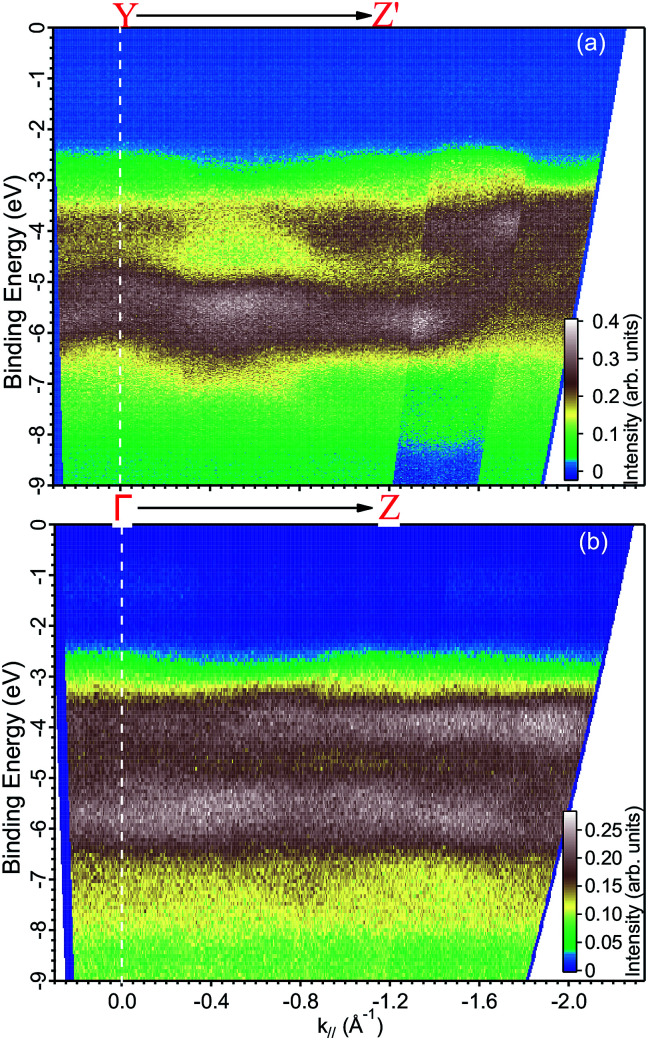
ARPES maps measured with a photon energy of (a) 35 eV along the YZ′ direction and at (b) 29 eV along the ΓZ direction.

The knowledge of the inner potential allowed us to fix the photon energy for the angle-dependent scans to map the bands, presented in Fig. 6, along the ΓZ direction and the one perpendicular to it.

**Fig. 7:**
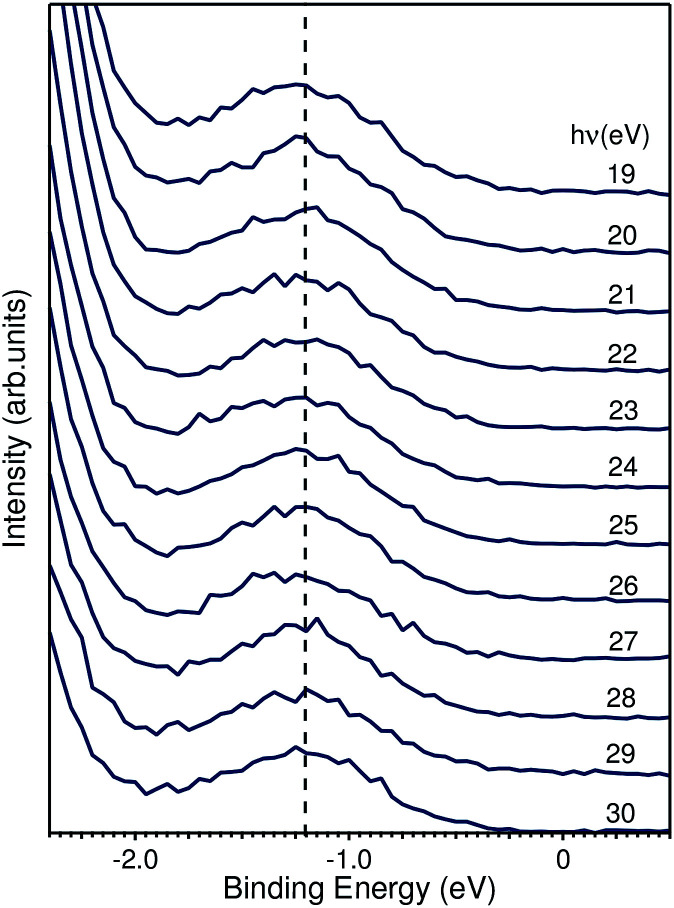
EDCs taken from Fig. 5a highlighting the binding energy region within the fundamental band gap. The peak shown has a binding energy of 1.2 eV and is photon-energy independent.

Finally, we present photoemission data measured in the energy region within the band gap. As seen in the EDC plot of Fig. 5 and in Fig. 7 hereafter, a dispersionless electronic state within the gap has been found at a binding energy of about 1.2 eV. A *k*-perpendicular dispersion is ruled out by the data in Fig. 7, while in-plane dispersions are also absent, as shown in the angular maps in Fig. 6 and ESI Fig. 1.† This electronic state is completely localized in real space and its possible origin is discussed below.

## 4 Discussion

The electronic structure of BiVO_4_ is complex because of many inequivalent atoms in the unit cell, which contains four unit formulas of the material. The major features can, however, be analyzed qualitatively by a comparison with calculations of the DOS.^11,14^ In these works, as well as in many other oxide systems, the oxygen states constitute the valence band, the vanadium states are unoccupied and a minor contribution from the bismuth 6s appears right below the valence band maximum. Ref. 11 published the DOS in a wide energy range and with this it is possible to attribute the main experimental peak at −5.5 eV (in Fig. 5) to a mixture of oxygen and vanadium orbitals, while the second-strongest peak at −3.5 eV derives purely from oxygen.

The experimental data readily show a dispersion, which is as large as 2 eV at some locations of *k*-space, for example in Fig. 6 at a binding energy around 3 eV and at parallel momentum vectors between 0.3 and 0.6 Å^−1^. To fully characterize the band dispersion, a calculation of the band structure would be necessary, but it is not available because all theoretical publications are done in the standard unit cell. We therefore welcome calculations done for the non-standard cell and a detailed a comparison to the experimental data to establish the validity of the theoretical approach.

We based the discussion of the peak in Fig. 7 in the band gap on the experimental observations that it has a binding energy of 1.2 eV. The lack of dispersion, neither in *k*_⊥_ (as shown in Fig. 7) nor *k*_∥_, as can be seen in Fig. 6, is a strong indicator that this is not a surface state. No dispersion rather indicates a strong localization in real space, which we attribute to the formation of a polaron state, *i.e.* the coupling between the hole (remaining in the solid after the photoelectron excitation) and the lattice degrees of freedom. Polaron formation has been first theoretically considered by Kweon *et al.*,^14^ who by hybrid density-functional theory calculated the band structure of charge-neutral and of electron-doped BiVO_4_. In the calculations for the electron-doped system, one extra peak emerges in the band gap, while the rest of the band structure remains virtually unchanged. The peak is qualitatively in the middle of the gap and the orbital decomposition shows that it is of mixed character with a prevalence of vanadium over oxygen. Experimentally, polaron formation was found to be responsible of the temperature dependence of the thermodynamic observables of BiVO_4_ (ref. 16) and a time-resolved THz spectroscopy study could measure in real time the polaron build-up and measure its activation energy.^17^

Attributing a polaron character to the peak in the gap has a number of consequences, the first of which is the determination of the fraction of Hartree–Fock exchange *α* needed to correctly reproduce the BiVO_4_ properties in theoretical calculations. As it can be inferred from Fig. 2 of ref. 14, the theory gives an almost linear dependence of the band gap size and the energy distance of the polaron peak form the valence band maximum as a function of *α*. The value of the gap alone can fix *α*, but the theoretical values of the bandgaps are for many systems lower than the experimental ones, a common problem, which can be ascribed to the local approximations in density-functional theory. Taking relative values can improve the comparison between theory and experiment and, using the notation of ref. 14, we can estimate the *α* using the following experimental values: Δ*E* = 1.15 eV and *E*_g_ = 2.55 eV, which imply Δ*E*/*E*_g_ = 0.45 and *α* ≈ 17%. This number could be used to calculate the band structure of BiVO_4_ and to compare them to our experimental bands. In ref. 15 a value of about 290 meV for the activation energy of the polaron was found, which is very close to the value one would obtain for *α* = 15%, as given in Fig. 3 of ref. 14. The latter value is very near to the value independently obtained by our ARPES experiments, which were also proposed in ref. 14.

**Fig. 8:**
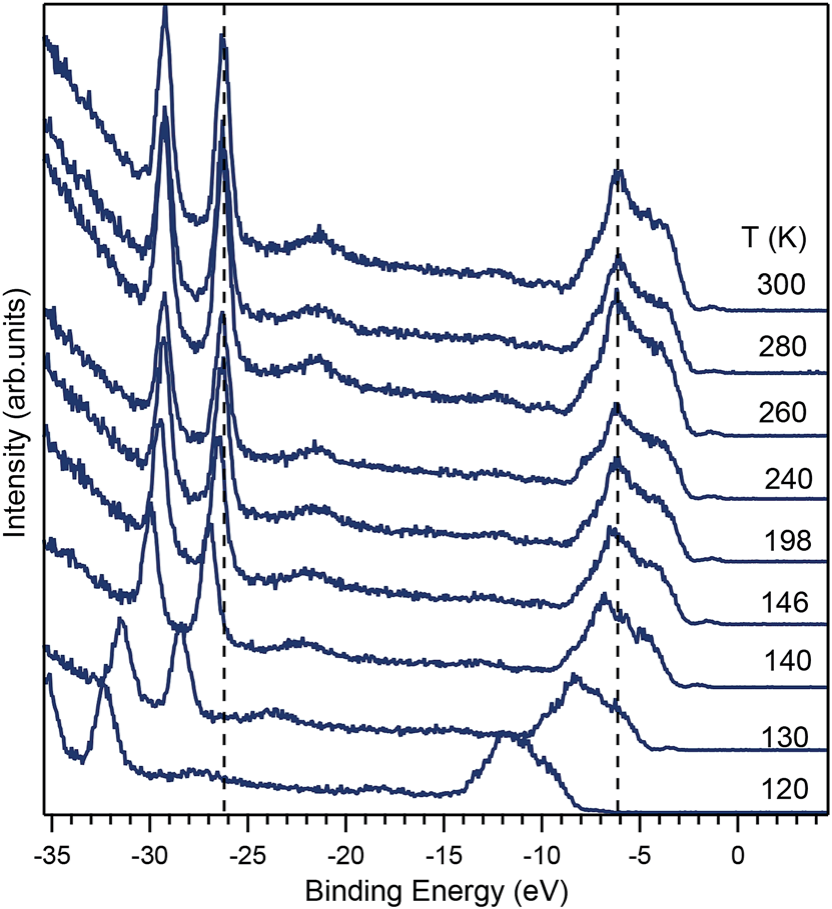
Temperature dependence of the photoemission spectra in BiVO_4_ taken with a photon energy *hν* = 50 eV at the temperatures indicated in the figure. The vertical dashed lines indicate the reference positions of the strongest peak in the valence band and of the Bi 5d_3/2_ peak at room temperature. When the temperature is reduced, the spectra shift to higher binding energies, an indication that the sample resistivity has increased.

Further evidence for polaron formation in BiVO_4_ is the temperature dependence of transport data in ref. 16. In this work it has been pointed out that the polaron formation is anisotropic and strongly temperature-dependent. While focusing on temperatures higher than 300 K, the authors state that the data are compatible with those of ref. 15, which, on the other had, explore also the low-temperature regime. The low-temperature data of ref. 15 show clearly a strong increase in the electrical resistivity upon a reduction of the temperature, which was explained by the formation of small polarons in the material. We attempted temperature dependent ARPES experiments and observed a strong increase in the resistivity of the samples upon cooling. As shown in Fig. 8, the samples charge up strongly, a sign that its resistivity has increased. For a detail view of the in-gap state evolution with temperature, see ESI.†

The charge-up effect occurs when the resistivity of the sample is high enough to hinder the flow of charge from ground to the sample, which is needed to neutralize the outflow of photoelectrons excited by the incoming photons. When this happens, a positive electric field (whose magnitude is determined by the balance between the photon intensity and the sample resistivity) builds up around the sample. The photoelectrons reaching the detector can overcome the electric field due to charge-up, but lose kinetic energy in doing that. Therefore, upon a resistivity increase, the whole spectrum appears shifted to high binding energies. Charge-up is thus not an intrinsic shift of the chemical potential, but solely an effect owed to a high sample resistivity.

The data in Fig. 8 indicate such a charge-up also due to a strong increase in the sample resistivity, which is qualitatively consistent with the behavior found in ref. 15. It was not possible to measure resistivity *in situ* during the ARPES experiment, but the observation that the spectrum does not shift between 300 K and 200 K, shows that the resistivity is low enough (for the used photon flux) for the sample to be charge-neutral. At *T* = 146 K a shift of about 0.5 eV is observed, while at 140 K, the shift already amounts to 1 eV and at 120 K it is 5.9 eV. At even lower temperatures, no experiment was possible, anymore. While it is tempting to compare the temperature dependence of the charge-up shift to the resistivity data, the comparison is not reliable, because the sample was illuminated by the UV beam during the experiment, a condition leading to the generation of photoexcited carriers, which are not present in a normal transport experiment. Thus, the temperature-dependent ARPES data show that the charge transport abruptly decreases below a specific temperature that depends on the doping. For the 1% of doping of our crystal, this specific temperature is about 140 K, but the latter will be certainly higher for lower doping levels.

With the information at hand, the explanation of the change in the conductivity at temperatures around 150 K is not unique. One possibility is derived from the data of Fig. 8, which shows that a change of 146 K to 120 K induces a complete charge-up of the system. This temperature difference corresponds to an energy scale of about 2.2 meV, which is a small and well below the experimental resolution, but is of the same magnitude as the change in the polaron activation energy due to a change in the lattice parameter predicted in ref. 14. Another possibility that could explain the charge-up effect is the temperature-dependent ionisation of donor defects, which could start to freeze below 150 K and thus reduce the conductivity to such low levels that the samples charges up. To obtain a qualitative estimation of the sample resistivity, we considered that the energy shift due to the charge-up is of the order of 1 eV, while the sample current was of the order of the nanoampere, implying that the sample under illumination had a resistivity of the order of the GΩ, a value that is in good agreement with the resistivity data of Rettie *et al.*^16^ The value for the resistivity that we obtained is an intrinsic one, since it remained constant when the beam intensity was (intentionally) changed. Moreover, we ensured that the photon beam spot on the sample remained at the same position for every temperature by readjusting the sample position. A clear distinction between polaron (de)activation and donor ionisation could in principle be possible by detailed analysis of the conductivity as a function of temperature, yet ARPES is unsuitable for this and we refer to the literature here.^16^

To clarify the origin of the in-gap peak, we investigated its dependence on the photon energy, since this method can reveal the orbital character of the peak. One common possibility are oxygen vacancies usually originating peaks in the gap of oxide compounds.^29,30^ A second one is that the Mo dopants form a peak in the band gap, as suggested by Jovic *et al.*,^31^ who showed by resonant photoemission experiments on BiVO_4_ doped with 0.6% Mo and 0.3% W that the peak in the gap actually stems from the doping atoms, but it resonates at the vanadium L edge, indicating a strong hybridization with vanadium. We extracted from the data in Fig. 5 the intensities of the strongest feature at *E*_B_ = 5.8 eV, considered as representative for the oxygen, and the intensities of the peak at *E*_B_ = 1.2 eV and took their ratio, *i.e.*
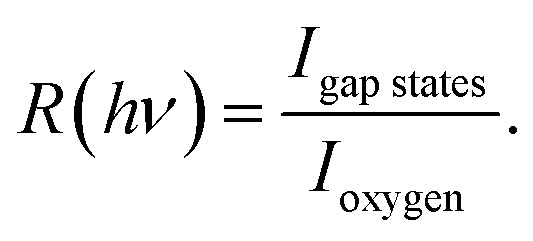
 Our hypothesis is that if the gap state originates from molybdenum, then *R*(*hv*) must depend on the photon energy approximately as the tabulated values.^32^

**Fig. 9:**
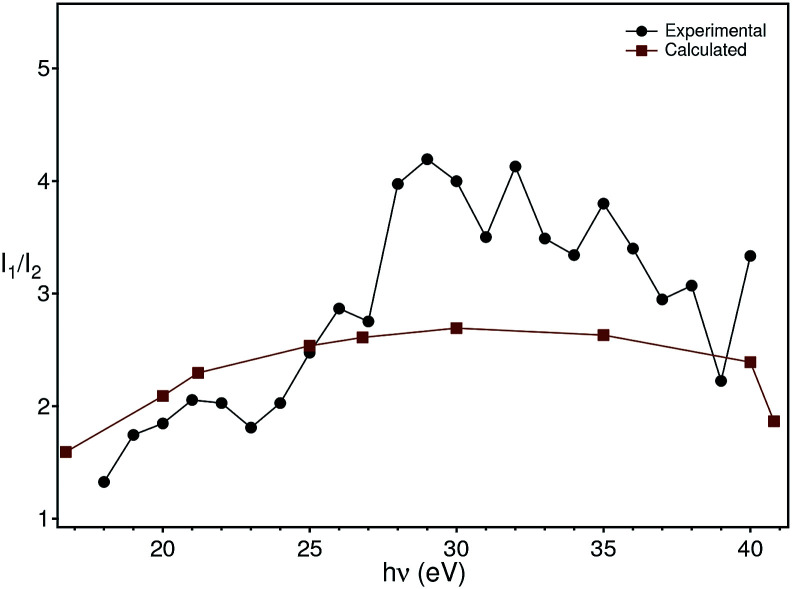
Comparison of the theoretical photon energy-dependent ratio (red) between the molybdenum and oxygen cross sections with the experimental ratio (black) between the peak intensity at −5.8 eV and −1.2 eV extracted from Fig. 5.

The theoretical intensities have been obtained by multiplying the values of the cross sections by the (small) correction of the angular distribution due to the energy dependence of the asymmetry parameter.^27^ The ratios of theoretical intensities and experimental peak ratios are compared in Fig. 9 after multiplying the experimental intensities with a factor of 100 to account for the molybdenum concentration in the material. The agreement is quantitatively good with the maximum of the cross section ratio at a photon energy of 33 eV present both in the theory and the experimental data.

Although the comparison between the experimental and theoretical intensity ratios is quantitatively reasonable, the approximations in the theoretical calculations and the finite acceptance angle of the electron analyser could, in principle, be the origin of the discrepancies observed at high energies in Fig. 9. In fact, the spectra were taken by integrating the intensities over a small angle around the normal emission direction. On the other hand, the calculations have been carried out for isolated atoms, which do not disperse, contrary to the experimental bands (especially the oxygen one). While these two aspects forbid a better comparison between theory and experiment, they nevertheless do not invalidate the assignment of the in-gap peak to polarons which involve electrons stemming from molybdenum. An argument supporting our analysis is that the assignment of the in-gap peak to Mo-related electrons is made comparing the ratio of intensities, whose term are photoemission matrix elements between different initial states (oxygen and molybdenum bands), but the same final state. The latter is actually the same for the two matrix elements, since the oxygen and molybdenum states binding energies are only a few eV from each other, *i.e.* they are very close to each other. Furthermore, due to backfolding of final state bands to the first BZ, practically a continuum of final states is formed for the photon energies applied here. Thus, the intensity ratio is to a good extent insensitive to the final state that the electron ends into and also does not make use of the free-electron final state approximation.

Based on this energy dependence of the cross sections, we conclude that the states in the gap originate from the molybdenum impurities. We thus model the doping and polaron formation in the following way: in BiVO_4_ the Mo (randomly) substitutes vanadium occupying the lattice position of the latter. Mo is, however, not isoelectronic to V, but has one extra electron acting as a donor, n-doping BiVO_4_ and placing the Fermi level near the conduction band bottom. The extra electron, stemming from Mo, hybridizes then with the vanadium and oxygen electrons and couples to the lattice originating strongly localized polarons, as observed in our ARPES experiments. Since polaron formation is a dynamical effect, it is not possible here to state that the 1% Mo reduces the band gap by creating statically localized states. Nevertheless, the macroscopic transport and optical properties of the material are completely changed by the doping: our pure BiVO_4_ single-crystals are semitransparent, pink-orange coloured and highly insulating, while those doped with Mo are metallic lustrous, not at all transparent and conductive, at least at room temperature. This is a coherent explanation of the contribution of the Mo electrons to the photoionization cross section (Fig. 9), of the resonant photoemission results^31^ and validates the theoretical prediction of a polaron state forming upon n-doping.^14^

The formation of polarons around the lattice sites where Mo sits is also important since the small polarons are small in BiVO_4_, *i.e.* the lattice distortion they cause is small and localized around the position of the dopant. In a photoelectrochemical cell the photoanode must come in contact with water to reduce it and consequently a surface barrier is created in the semiconductor. Because of the local lattice distortion due to polarons, a local modification of the surface barrier can occur. Since the doping is random at the atomic scale, Mo defects can inhomogeneously trap charge at the interface and considerably alter the transport of the holes from the semiconductor to water, therefore reducing the performance of the photoelectrochemical cell. Scanning tunnel microscopy experiments on TiO_2_(101) have actually observed that around subsurface defects polarons form and can get self-trapped by their strong local field and even their distribution can be modified by voltage application.^33^ This does not imply that the same must and will occur in BiVO_4_, but it is certainly a phenomenon that is worth being investigated and its importance assessed when designing a water splitting device.

The formation of polarons has been studied in great detail in oxide semiconductors, for example in manganites and copper-based high-temperature superconductors. In these materials however, the doping leads to metallic systems with bands crossing the Fermi level. The coupling to polarons flattens and broadens these bands while the metallic character of the material is partially retained. In these systems, the ARPES spectra may show the characteristic peak-dip-hump lineshape, which is the signature of the coupling between of the electrons with the polarons. However, in BiVO_4_, the 1% doping is not sufficient to bring the semiconductor in the degenerate partially metallic state, thus a peak-dip-hump lineshape cannot be expected. Only an isolated peak in the gap can be expected, as shown by our ARPES data and also indicated by the theoretical calculations.^14^

## 5 Conclusion

In our investigation, we have determined the experimental band structure of Mo-doped BiVO_4_, grown by the Czochralski method, with ARPES along several high-symmetry directions of the Brillouin zone. The bandwidth of the valence band was found to be 4.75 eV, which is smaller by 0.7 eV than the reported calculated value. The ARPES data compare well with the published calculations concerning the bandwidth and the orbital character of the states. Also the experimental band gap is in good agreement with the theoretical one, if the underestimation of the gap by the theory is considered.

We made the important observation that in the fundamental gap there is an electronic state that does not disperse in all three dimensions of the reciprocal space, which indicates a complete localization in real space, suggesting that it is not a surface state. We attribute this peak to localized polarons and by comparing the ratio between the in-gap peak and the width of the band-gap we find that a mixing parameter *α* = 17% has to be used in hybrid density-functional calculations to correctly describe the electronic properties of BiVO_4_.

By analyzing the temperature dependence of the ARPES spectra a strong charge-up effect has been found below 150 K, which is attributed to the polarons that strongly limit the transport of electrons and are therefore the origin of a strong increase in the sample resistivity. From the shift in the ARPES spectra we could determine that the polaron activation energy is severely affected by changes in the unit cell volume. Though these changes are in the meV range, their effect is very large.

Finally, a thorough analysis of the photon energy dependence of the experimental in-gap and top-of-valence band peak intensities lets us understand the chemical character of the in-gap peak and the draw a coherent interpretation of the ARPES and transport data as well as of the theoretical calculations.

## Conflicts of interest

There are no conflicts to declare.

## Supplementary Material

RA-009-C9RA01092K-s001
