# Supplementary material for: The electronic structure and the formation of polarons in Mo-doped BiVO4 measured by angle-resolved photoemission spectroscopy
Source: RSC Adv. 2019 May 17;9(27):15606–14. doi: 10.1039/c9ra01092k (PMC9064338; doi:10.1039/c9ra01092k)
Supplement: RA-009-C9RA01092K-s001 [file RA-009-C9RA01092K-s001.pdf]

Electronic Supplementary Material (ESI) for RSC Advances.
This journal is © The Royal Society of Chemistry 2019

Electronic Supplementary Information for: *"The electronic structure and the formation of polarons in Mo-doped $BiVO_4$ measured by angle-resolved photoemission spectroscopy"*

Mansour Mohamed,[1,2] Matthias M. May,[3] Michael Kanis,[3,4] Mario Brützam,[5] Reinhard Uecker,[5] Roel van de Krol,[3] Christoph Janowitz,[1] and Mattia Mulazzi[1,6]

[1] *Institut für Physik, Humboldt-Universität zu Berlin, D-12489 Berlin, Germany.*

[2] *Department of Physics, Faculty of Science, Assiut University, 71515 Assiut, Egypt.*

[3] *Institute for Solar Fuels, Helmholtz-Zentrum Berlin, D-14109 Berlin, Germany.*

[4] *OUT e.V., D-12555 Berlin, Germany.*

[5] *Leibniz-Institut für Kristallzüchtung, D-12489 Berlin, Germany.*

[6] *Institute Functional Oxides for Energy-Efficient IT, Helmholtz-Zentrum Berlin, D-14109 Berlin, Germany*

In ESI Fig. 1 we show the dispersion measured using the second angular degree of freedom of our manipulator. For these measurements, the angular step is about 1°, measured by tilting the sample in the plane perpendicular to the analyzer slit.

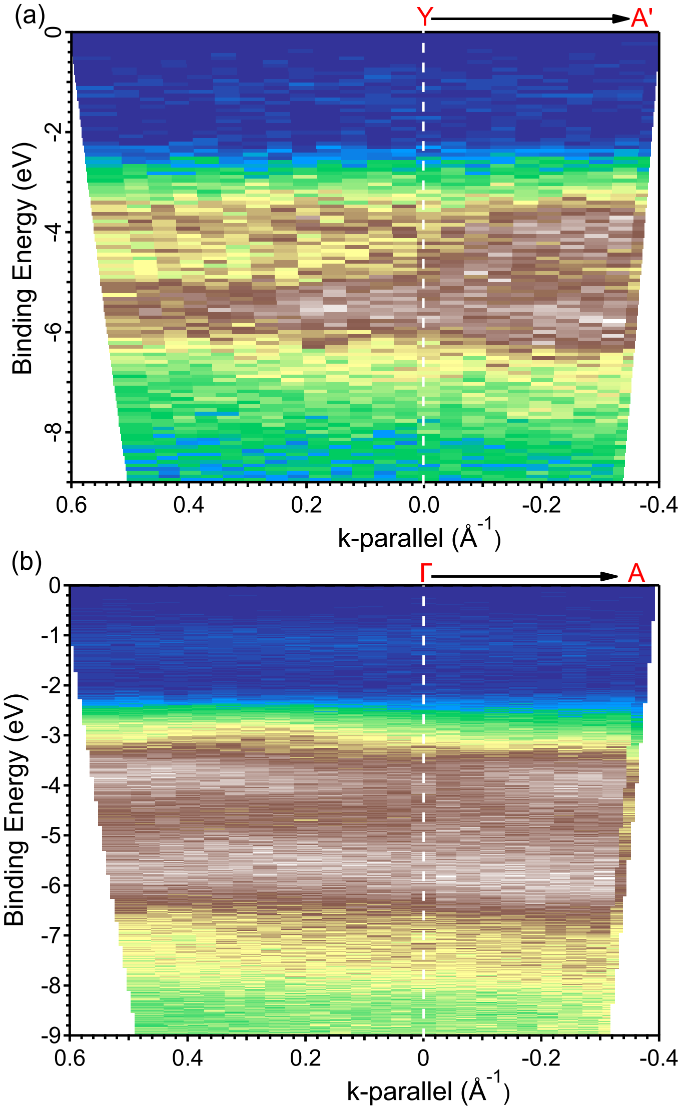

FIG. 1. ARPES maps measured perpendicular to the (a) YZ direction with a photon energy of 35 eV and (b) the ΓZ' direction with a photon energy of 29 eV. Since $BiVO_4$ has a monoclinic cell, the wavevectors spanned are approximately parallel to the YA' and and ΓA' directions, respectively (see main text).

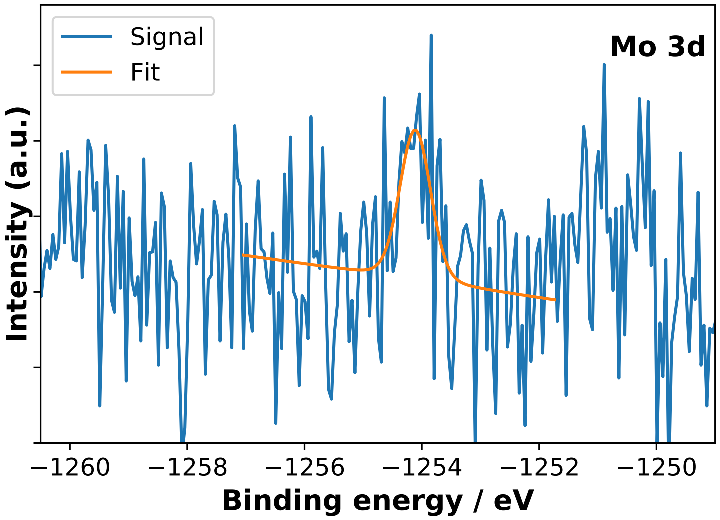

FIG. 2. X-ray photoelectron spectroscopy around the Mo 3d signal with a monochromated Al K_α source.

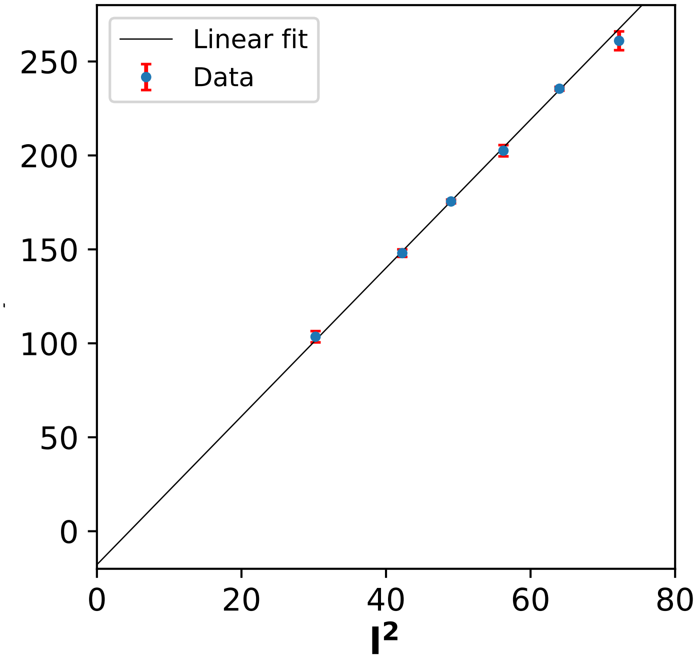

FIG. 3. Madelung graph used for the determination of the inner potential, U_0, by means of a least square fit according to $E = l^2 c - U_0$, where c is a constant and E the energy, at which a maximum of the primary Bragg peak was observed (including half-order peaks).

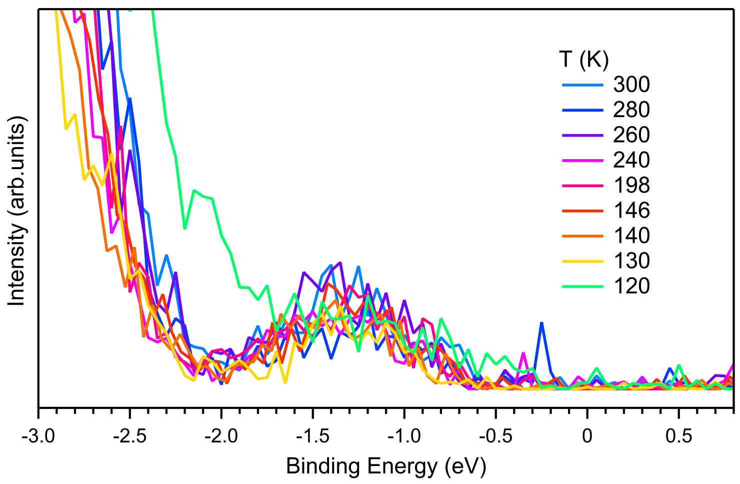

FIG. 4. Detail view of the in-gap signal from Fig. 8 (of the main manuscript) as a function of temperature. The intensity was normalised with respect to the total photon flux.
